# Complete Genome Sequence of *Coriobacteriaceae* Strain 68-1-3, a Novel Mucus-Degrading Isolate from the Swine Intestinal Tract

**DOI:** 10.1128/genomeA.01143-15

**Published:** 2015-10-08

**Authors:** T. Looft, D. O. Bayles, D. P. Alt, T. B. Stanton

**Affiliations:** a Food Safety and Enteric Pathogens Research Unit, National Animal Disease Center, Agricultural Research Service, United States Department of Agriculture, Ames, Iowa, USA; b Infectious Bacterial Diseases Research Unit, National Animal Disease Center, Agricultural Research Service, United States Department of Agriculture, Ames, Iowa, USA

## Abstract

A novel *Coriobacteriaceae* bacterium (strain 68-1-3) was isolated from the ileum of the swine intestinal tract using a selective mucus-based medium. Here we present the finished genome sequence for the swine commensal, totaling 1.97 Mb in size.

## GENOME ANNOUNCEMENT

A heterogeneous distribution of bacteria exists along the swine intestinal tract suggesting local adaptations that may influence animal health and disease (1). *Coriobacteriaceae* and other members of the *Actinobacteria* phylum are common members of the mammalian intestinal microbiota (2) including known beneficial microbes (3). Other members of the *Coriobacteriaceae* family can utilize mucin as a growth substrate, suggesting mucus degradation is a conserved trait (4, 5). A novel *Coriobacteriaceae* bacterium, strain 68-1-3, was isolated from the distal ileum of the swine intestinal tract, in accordance with the National Animal Disease Center Animal Care and Use Committee guidelines, using a minimal medium, supplemented with hog gastric mucin (6). The closest cultured relative of 68-1-3 is *Adlercreutzia equolifaciens* DSM 19450, which shares 94% 16S rRNA gene sequence identity. Strain 68-1-3 shows important genomic differences from *A. equolifaciens* including the absence of “giant genes,” fewer predicted protein-coding regions, and an overall smaller size (7).

High-quality genomic DNA was extracted using the Marmur method (8) from a 1-liter culture of 68-1-3 grown in modified M2GSC medium (9), with depleted rumen fluid substituted for clarified rumen fluid (10). Sequencing was performed using both Illumina HiSeq (Illumina, Inc., San Diego, CA, USA) and Roche FLX-Titanium chemistry (Roche Diagnostics, Branford, CT, USA). Libraries were prepared according to manufacturer’s directions. A fully closed genome consisting of one chromosome was assembled using MIRA v4.0.2 (11) coupled with information derived from draft assemblies created using the Roche gsAssembler v2.8. The primary MIRA assembly was a *de novo* hybrid assembly comprised of Roche FLX shotgun sequencing reads, Roche FLX 2.3-kb mate-pair library reads, and Illumina 7.9-kb mate-pair library reads (2 × 150 bp, rapid mode). The assembled and closed genome had 86.5× average coverage, with the FLX data providing 40× and the Illumina data providing 46× of the total genome coverage. Roche gsAssembler assemblies used only Roche sequencing data obtained from GS FLX shotgun and GS FLX Titanium 2.3 kb mate-pair sequencing reads. Genome editing was performed using Gap5 from the Staden Package (12) 

Genome annotation and statistics were generated with the NCBI Prokaryotic Genomes Automatic Annotation Pipeline (13). The complete genome of strain 68-1-3 is 1,967,093 bp, encoding 1,723 predicted genes, including: 6 rRNA genes, 48 tRNAs, and 12 pseudogenes. The G+C content of the genome is 63.6%. The closest “neighbor,” identified by whole genome comparison using the RAST (Rapid Annotation using Subsystem Technology) web tools and database, was the human intestinal isolate, *Eggerthella lenta* (DSM 2243) (14). Strain 68-1-3 was quite divergent from *E. lenta*, (92% 16S rRNA gene sequence identity) and the strain 68-1-3 genome is 1.2 Mb smaller and contains 1,471 fewer genes. These genome-wide differences and low 16S rRNA gene sequence identity with *A. equolifaciens* indicate that strain 68-1-3 is likely a newly discovered genus within the *Coriobacteriaceae* family found inside the swine intestinal tract.

### Nucleotide sequence accession number.

The complete genome entry has been deposited in GenBank under the accession number NZ_CP009302.
